# Effectiveness of a Low-Calorie Diet for Liver Volume Reduction Prior to Bariatric Surgery: a Systematic Review

**DOI:** 10.1007/s11695-020-05070-6

**Published:** 2020-11-02

**Authors:** Marleen M. Romeijn, Aniek M. Kolen, Daniëlle D. B. Holthuijsen, Loes Janssen, Goof Schep, Wouter K. G. Leclercq, François M. H. van Dielen

**Affiliations:** 1 Department of Surgery, Máxima Medical Center, De Run 4600, 5504 DB Veldhoven, The Netherlands; 2 Faculty of Health, Medicine and Life Sciences, Maastricht University, Universiteitssingel 40, Maastricht, 6229 ER The Netherlands; 3 Department of Sport Medicine, Máxima Medical Center, De Run 4600, Veldhoven, 5504 DB The Netherlands

**Keywords:** Bariatric surgery, Low-calorie diet, Liver volume, Preoperative diet

## Abstract

An energy-restricted diet is often prescribed before bariatric surgery to reduce weight and liver volume. While very-low-calorie diets (VLCDs, 450–800 kcal per day) have shown to be effective, the effectiveness of low-calorie diets (LCDs, 800–1500 kcal per day) is less obvious. The objective of this systematic review was to elucidate the effectiveness of LCD on liver volume reduction in patients awaiting bariatric surgery. Eight studies (*n* = 251) were included describing nine different diets (800–1200 kcal, 2–8 weeks). An LCD was effective in liver volume reduction (12–27%) and weight loss (4–17%), particularly during the first weeks. The LCD showed an acceptable patients’ compliance. Based on these findings, an LCD (800–1200 kcal), instead of a VLCD, for 2 to 4 weeks should be preferred.

**Supplementary Information:**

The online version contains supplementary material available at 10.1007/s11695-020-05070-6.

## Introduction

Bariatric surgery is considered the most effective treatment for severe obesity as it promotes long-term weight loss and reduces or controls obesity-related comorbidities [1]. The incidence of short-term life-threatening complications is considered relatively low (1–5% for anastomotic leakage and bleeding) [2, 3] but depends on the patients’ comorbidities and technical difficulties that are encountered during surgery. In obese patients, technical difficulties are related to increased abdominal wall thickness, increased visceral adiposity and the presence of an enlarged liver. All these factors may contribute to reduced intra-abdominal space, reduced freedom of surgical movement and limited exposure of the gastric cardia, making the surgery technically more challenging and potentially resulting in complications [4, 5]. Up to 90% of candidates for bariatric surgery have nonalcoholic fatty liver disease (NAFLD) characterized by an enlarged and fatty liver [6]. An enlarged left liver lobe complicates the approach to the gastroesophageal junction and results in an increased risk of bleeding upon surgical manipulation since the NAFLD liver is more vulnerable [5].

For these reasons, it is imperative that a patient lowers weight and liver volume prior to bariatric surgery. In order to do so, an energy-restricted diet is routinely prescribed. There is however a lack of consensus regarding the optimal composition of this diet. A very-low-calorie diet (VLCD) and a low-calorie diet (LCD) are both popular hypocaloric diets that are widely advised [5, 7, 8]. A VLCD is generally defined as an intake of 450–800 kcal per day, while an LCD implies 800–1500 kcal per day [9, 10]. The duration of very-low-calorie diets (VLCDs) varies between 10 and 63 days, and the consistency varies between exclusively liquid meal replacements or a combination of liquid meal replacement and food meals [4, 5, 10]. In 2017, a systematic review showed that a VLCD was effective in liver volume reduction (5–20%, mean 14%) [10]. Several studies indicate that an LCD may also be effective [10–12], but a similar systematic review has not been performed yet.

When prescribing a VLCD and LCD, there are potential risks that need to be considered. One of the two prevailing risks is that the diet may turn the body into a catabolic state leading to lean body mass (LBM) loss [13]. A decreased LBM could negatively impact energy balance, functional capacity and cardiovascular health [14, 15], which may impede recovery after bariatric surgery [16]. Secondly, the patient may experience symptoms related to the catabolic state like fatigue, headache and nausea compromising the compliance and acceptability of the diet [17]. How these risks relate to the level of dietary restriction is unclear, but it is intuitive that the risks are larger in a higher degree of caloric restriction. This leads to a substantial doubt as to whether a VLCD should be the preferable diet.

The purpose of this systematic review was to evaluate the literature on the effect of an LCD on liver volume reduction in patients awaiting bariatric surgery. If an LCD would result in sufficient liver volume reduction, this diet could be a preferable alternative for the commonly prescribed VLCD [7].

## Methods

This review complies with the recommendations of the Cochrane Handbook for Systematic Reviews and Interventions [18] and was recorded according to the PRISMA systematic review guidelines [19]. The review was registered at PROSPERO as registration number CRD42020176838.

### Systematic Literature Search

The systematic search was conducted on February 13, 2020, and was performed in three online databases: MEDLINE (PubMed Legacy), EMBASE (Ovid), and The Cochrane Library. The search was restricted to articles published in English and Dutch. There was no restriction regarding the date of publication. Keywords in the search strategy included [low calorie diet] and [bariatric surgery] and their synonyms. The full search strategies for all databases can be found in Supplementary Table 1. Reference lists of identified articles were manually screened to retrieve articles that might have been missed. The authors were contacted by email if no full text was available online.

### Eligibility Criteria

This review included randomized controlled trials (RCTs) and observational studies. Inclusion criteria were (1) prescription of low-calorie diets (LCDs) containing 800 to 1500 kcal/day with a duration of at least 5 days and up to 3 months, (2) patients with a BMI ≥ 35 kg/m^2^ and selected for bariatric surgery, (3) assessment of liver volume by magnetic resonance imaging (MRI), computed tomography (CT) or ultrasound, and (4) caloric intake obtained from standardized meals or more than 75% from prescribed meals with dietary compliance controlled by urinary ketone. Food-based self-selection or energy prediction based on food recalls was excluded. Articles were excluded if they were designed as animal studies or as reviews, letters to the editor and conference abstracts.

### Study Selection

Database searches were imported into Endnote X9 to manage references and support identification of duplicates. Titles and abstracts were screened on relevance. Full texts were obtained for clarification of eligibility criteria. Excluded studies and the reason for exclusion were recorded.

### Data Extraction

Data abstraction was performed by two reviewers (AK, MR) who used pre-defined forms for the following study characteristics: authors’ names, publication year, country, study design, sample size, gender, mean age, mean BMI, kcal/day, duration and composition of the diet. Additionally, information about liver volume, weight, body composition, tolerance and acceptability of the diet, surgical complexity, complications and biochemical- and clinical parameters was extracted.

### Outcome Parameters

The primary outcome was liver volume reduction (total or left liver lobe) by LCD prior to bariatric surgery. Secondary outcomes were differences in weight and body composition, represented in means. Additional outcomes were tolerance and acceptability of the diet, surgical complexity, complications and biochemical- and clinical parameters. Standard deviations were extracted if available. If only pre- and post-data was provided, a percentage was calculated from these data points.

### Quality Appraisal

The methodological quality of the included studies was assessed using the Cochrane risk of bias tool [20] for randomized controlled trials (RCT) and a modified Methodological Quality Checklist as described by Downs and Black [21] for non-RCTs. For the Cochrane risk of bias tool, studies were classified as “high” risk of bias if two or more indications of “high” risk of bias were classified. Furthermore, studies with three or more indications of “unclear” risk of bias were classified as “moderate” risk of bias, while studies were classified as “low” risk of bias if they had four or more indications of “low” risk of bias. Downs and Black’s checklist was modified to increase suitability as no control group was included in the non-RCTs. An overview can be found in Supplementary Table 2. A score of 25–27 points was considered excellent, 19–24 was considered good, 14–18 was considered fair and ≤ 13 was classified as poor study quality. Two reviewers (AK, MR) critically assesed the quality of the studies independently. Forthcoming discrepancies were resolved in accordance with both reviewers.

## Results

**Fig. 1:**
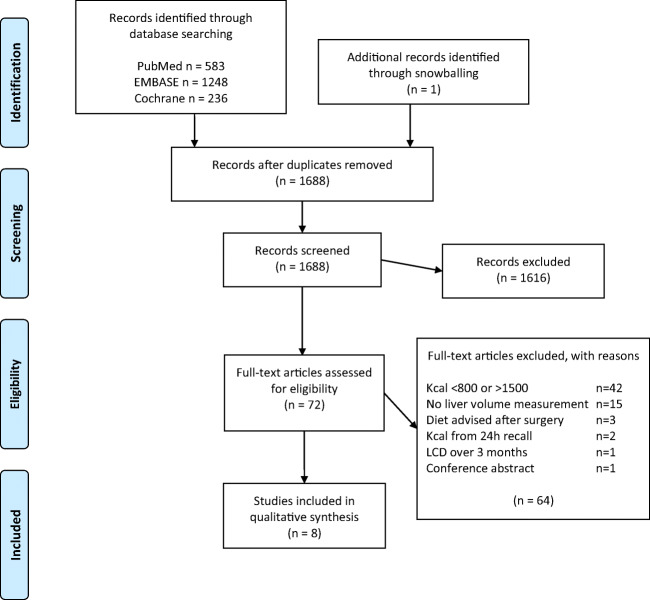
PRISMA flow diagram showing selection of articles

The search retrieved a total of 2067 records. An additional manual check of reference lists resulted in the addition of one study. After removing duplicates, 1688 studies remained. After screening the titles and abstracts on relevance, 1616 of the 1688 articles were excluded. Full-text reading of the remaining 72 articles resulted in the inclusion of eight eligible studies (Fig. 1).

### Study Characteristics

Of the eight included studies, three studies were RCTs [12, 22, 23] and five were observational studies [4, 11, 24–26]. One study described two LCDs; both were included in this review [12]. A total of 251 patients, with an age of 34–46 years, were included. A control group to assess liver volume was included in three studies, with two studies receiving no dietary intervention [22, 24] and in one study omega 3 supplementation on top of a 2000 kcal diet [23]. Six studies assessed total liver volume [4, 11, 12, 23, 25, 26], while only two studies exclusively assessed left liver lobe volume [22, 24] (Table 1). Nine LCDs were included with varying dietary characteristics among eight studies. Energy intake ranged from 800 to 1200 kcal daily with heterogeneity in diet composition and consistency (Table 1). The duration of the diet ranged from two to eight weeks with a median duration of four weeks.

**Table 1:** Summary of the study characteristics of the included studies

Year	Author	Country	Study design	Sample size (female gender)	Initial BMI (kg/m^2^)	Kcal per day and diet type	LCD duration (weeks)	Diet composition	Measurement left lobe and/or total liver
2019	Bakker et al. [23]	The Netherlands	Randomized controlled trial	26 (26)	41.4 (6)	800 Modifast + food	2	unknown	MRI Left lobe and total liver
2019	Chakravartty et al. [22]	United Kingdom	Randomized controlled trial	10 (10)	53.4 (45.1–61.7)	800 Cambridge milk diet	4	82 g CHO 61 g PRO 30 g FAT	Ultrasound Left lobe
2018	Contreras et al.* [12]	Spain	Randomized clinical trial	43 (29)	47.3 ± 5.3	800 Optifast	3	107 g CHO 73 g PRO 8 g FAT	CT Total liver
2018	Contreras et al.* [12]	Spain	Randomized clinical trial	41 (34)	47.2 ± 5.0	1200 Food + Optifast	3	166 g CHO 109 g PRO 12 g FAT	CT Total liver
2011	Edholm et al. [11]	Sweden	Prospective observational	15 (15)	42.9 ± 3.0	800–1100 Modifast	4	124 g CHO 59 g PRO 22 g FAT	MRI Total liver
2015	Edholm et al. [26]	Sweden	Prospective observational	10 (10)	41.7 ± 2.6	800–1100 Modifast	4	124 g CHO 59 g PRO 22 g FAT	MRI Total liver
2020	Ekici et al. [24]	Turkey	Retrospective observational	49 (32)	45.1 ± 4.4	1000 Unknown	4	Unknown high PRO	Ultrasound Left lobe
2013	González-Pérez et al. [4]	Mexico	Prospective observational	20 (17)	46.0 ± 5.3	800 Food	6	40 g CHO 68 g PRO 41 g FAT	CT Total liver
2015	Schiavo et al. [25]	Italy	Prospective cohort study	37 (0)	45.2 ± 4.9	1200 Food	8	141 g CHO 80 g PRO 35 g FAT	Ultrasound Left lobe and total liver

*Study included two low-calorie diets

Abbreviations: *LCD*, low-calorie diet; *BMI*, body mass index; *MRI*, magnetic resonance imaging; *CT*, computed tomography; *CHO*, carbohydrates; *PRO*, protein; *FAT*, fat; *Left lobe*, left liver lobe volume; *Total liver*, total liver volume

Values are expressed as mean, mean ± SD, mean (CI) or mean (range-range)

### Quality of the Studies

Two RCT studies [22, 23] scored a low risk of bias and one RCT study [12] scored a moderate risk of bias on the Cochrane risk of bias tool. All of the observational studies [4, 11, 24–26] scored a fair study quality on the Modified Methodological Quality Checklist as described by Downs and Black. Blinding of both participants and personnel, as well as external validity of subjects, lacked in most of the observational studies [4, 11, 24–26]. Blinding also lacked in one of the RCTs [12]. None of the observational studies [4, 11, 24–26] performed a power calculation based on liver volume reduction. Quality assessment of the included studies is presented in Supplementary Fig. 1 (for RCTs) and Supplementary Table 2 (for observational studies).

### Liver Volume Reduction

Left liver lobe volume showed a decrease of 11–29% [22–25] and total liver volume showed a decrease of 12–27% with a mean of 16% [4, 11, 12, 23, 25, 26]. Studies with a diet ranging between 2 and 4 weeks [11, 12, 22, 24, 26] showed a liver volume decrease of 11–23% (Table 2).

**Table 2:** Results of liver volume reduction and changes in body weight

Author	Change in liver volume	Change in weight	Additional liver measurement
Bakker et al. [23]	Total: − 12.7% LL: − 11.1%	BMI: − 4.6%	Surgeon scored 31% of the patients with an enlarged liver and 39% had a liver that was fatty with yellow discoloration
Chakravartty et al. [22]	LL: − 23% Control: − 2%	TWL: − 5.4% FM: 40.3% LBM: 59.7%	No change in fibrosis, shown by ARFI and APRI
			No difference in left liver lobe size, sharpness of liver edge, exposure of hiatus and diaphragm from control
Contreras et al. [12], 800 kcal	Total: − 15.6 ± 11.2%	TWL: − 5.8% FM: 53.8% LBM: 46.2%	Liver enzymes: increase in AST and ALT, and unaffected GGT
Contreras et al. [12], 1200 kcal	Total: − 12.3 ± 10.6%	TWL: − 4.2% FM: 49.1% LBM: 50.9%	Liver enzymes: unaffected AST and ALT, and decreased GGT
Edholm et al. [11]	Total: − 12%	TWL: − 6.1%	Intrahepatic fat decreased by 40% from 9.41 ± 6.17% to 5.53 ± 4.11%
			Surgeon’s perception: decreased left lobe size and better sharpness of liver edge and exposure of hiatal region compared to controls
Edholm et al. [26]	Total: − 18 ± 4%	TWL: − 6.5% FM: 71.2% LBM: 28.8%	Intrahepatic fat decreased by 51 ± 16%
			Liver volume reduction within the first 2 weeks, no further change afterwards
			Liver enzymes: unaffected AST and ALT
Ekici et al. [24]	LL: − 11.2% Control: 0.7%	TWL: − 4.4%	Not assessed
González-Pérez et al. [4]	Total: − 20.3%	EWL: 14.4 ± 5.9%	Liver volume reduction Week 0–2: − 22%; Week 2–4: − 13%; Week 4–6: +17%
Schiavo et al. [25]	Total: − 26.9% LL: − 29.1%	TWL: − 16.7% FM: 77% LBM: 23%	Liver enzymes: decreased GOT and GPT, and unaffected GGT

*ARFI*, acoustic-radiation force-impulse imaging; *APRI*, aspartate aminotransferase to platelet ratio index; *AST*, aspartate aminotransferase; *ALT*, alanine transaminase; *BMI*, body mass index; *EWL*, excess weight loss; *FM*, fat mass; *GGT*, gamma glutamyl transferase; *GOT*, glutamic oxaloacetic transaminase; *GPT*, glutamic pyruvic transaminase; *LBM*, lean body mass; *LL*, left liver lobe volume; *TWL*, total weight loss

### Weight Loss

Six of the eight studies reported the pre and post LCD weight [11, 12, 22, 24–26]. The weight loss ranged from 5.4 to 23.6 kg, corresponding with a percentage original body weight loss ranging from 4.2 to 16.7% with a median of 6.0% (Table 2). In the diets with a duration of 2 and 4 weeks, a body weight loss of 4.2–6.5% was observed [11, 12, 22, 24, 26].

### Body Composition

Four studies [12, 22, 25, 26] assessed body composition. Three studies [12, 25, 26] measured body composition by bioimpedance, while one study used dual-energy X-ray absorptiometry (DEXA) [22]. LBM accounted for 22.9–59.7% of the weight loss with a median of 50.9%. This implies that 40.3–77.1% of the weight loss was fat mass.

### Compliance and Tolerance of LCD

Six studies [4, 12, 23–26] recorded compliance and tolerance of the LCD. Compliance was measured in four different ways: (1) presence of ketonuria [24, 26], (2) the combination of presence of ketonuria with weight loss [4, 25], (3) formula sachets returned [12], and (4) unblinded patient interviews [23]. The studies reported a generally high compliance of 80–89% [4, 12, 24]. Tolerance was measured in three different ways: (1) questionnaires [4, 25, 26], (2) unblinded patient interviews [23], and (3) unknown assessment technique [12]. In general, the LCD was well tolerated, but some studies reported side effects like hunger, nausea, the feeling of wanting to chew, headache, diarrhea or constipation, and dizziness [4, 12, 23, 26].

### Surgical Outcomes and Complications

Mixed results on surgical complexity ratings were found. One study reported improvement of surgical complexity after LCD [11], while another study reported no change in surgical complexity [22]. Surgical duration decreased in one study [24], while two studies found no difference [11, 22]. No difference in incidence of complications was observed [12, 22–24] (Supplementary Table 3).

## Discussion

A VLCD is known to be effective in liver volume reduction (5–20%, mean 14%) according to a previously published systematic review including 140 patients [10]. However, it also results in negative side effects due to this extreme energy restriction. This systematic review identified eight studies with nine LCDs ranging from 800 to 1200 kcal. All studies demonstrated that an LCD was effective in reducing liver volume (12–27%, mean 16%).

The largest decrease in liver volume was observed when an LCD lasted for two to four weeks. Previously, Edholm et al. demonstrated that liver volume decreased during the first two weeks with 18 ± 6.2% and no further change afterwards [11]. Moreover, Gonzales-Perez et al. measured a decrease of 32% between baseline and week four after an LCD and a much smaller decrease (17%) between week four and six [4]. These findings are confirmed by Colles et al. who demonstrated that 80% of total liver volume reduction occurred in the first two weeks [27]. This overlapping data indicates that a dietary duration of two to four weeks is sufficient to induce liver volume reduction and should be preferred in clinical practice.

In order to assess whether a VLCD should be substituted by an LCD, it is important to evaluate the downsides including LBM loss. This study found that 51% of the weight loss was contributed to LBM loss rather than fat mass loss. When comparing this finding with a VLCD, previous research showed that this resulted in an even larger LBM loss (62%) [28]. This indicates that an LCD leads to less LBM loss, but there are some notes of caution hampering firm conclusions. This review reported a high variety in results with two studies that showed a LBM loss of 23–29% [25, 26] and three studies that showed a LBM loss of 46–60% [12, 22]. Moreover, three studies [12, 25, 26] measured body composition by bioelectrical impedance analysis which is prone to error [29]. In future research, it is important to realize that LBM preservation not only relies on dietary composition but also on physical activity [30]. Up to now, exercise has shown promising results in LBM preservation in patients awaiting bariatric surgery [31], though the effect on liver volume is unknown.

When evaluating the side effects, this study found that an LCD was well tolerated and that patients were highly compliant (80–89% compliance rate). Yet again, this data must be interpreted with caution because some studies determined compliance using subjective methods such as counting the returned empty formula sachets and interviewing patients in an unblinded manner. Additionally, the high compliance rate and few side effects might be explained by the relatively short period of energy restriction.

This study observed that perceived surgical complexity, duration of surgery and hospital stay were improved or remained the same, and that complication rate was unchanged. Previously, van Nieuwenhove et al. demonstrated that, in a single-blinded RCT, a two weeks lasting LCD reduced perceived surgical difficulty and 30-day complications, without affecting the duration of surgery [32]. Additionally, a Scandinavian study including over 22,000 patients showed that a weight loss of about 5% reduced the risk of overall postoperative complications in the range of 13–18% [33]. The inconsistencies between these findings and the findings of this review might be attributable to insufficient power, lack of blinding by the surgeon and different dietary approaches. Further RCTs are necessary to clarify the controversy of the effect of an LCD on surgical complexity and complications.

There are several limitations that should be considered when interpreting this systematic review. First, there was a large heterogeneity in terms of diet composition, diet duration and liver volume measurement. Second, different surgical techniques were used which may represent different populations. Third, the quality of the studies was limited with five observational studies being included. Fourth, a control group and blinding of assessor lacked in almost all of the studies which may have caused detection bias. Lastly, secondary outcomes were underpowered thereby possibly failing to detect differences.

In the future, it could be questioned if all patients will actually benefit from a universal LCD. Perhaps preoperative diets would be better in a personalized way, depending on what goals are being set by a multidisciplinary team. These goals could vary between patients with different BMI’s or comorbidities, for example reduction in liver volume or stabilization of glucose levels. It is warranted to perform new studies investigating the effect of LCDs in different study populations.

## Conclusion

This study demonstrates that an LCD is effective in reducing liver volume and weight. It is recommended that an LCD provides 800–1200 kcal per day and that it lasts for 2 to 4 weeks. Based on prior literature involving a VLCD, it appears that an LCD is even effective in liver volume reduction. Hence, an LCD should be preferred because, in this way, unnecessary excessive dietary restriction and subsequent downsides (e.g. LBM loss, side effects) can be countered. Further research should explore personalization of preoperative diets and focus on the effects of exercise on liver volume and LBM preservation in bariatric candidates.

## Supplementary information

ESM 1 (DOCX 68 kb)

## Abbreviations

BMI: Body mass index
LBM: Lean body mass
LCD: Low-calorie diet
NAFLD: Nonalcoholic fatty liver disease
RCT: Randomized controlled trial
TWL: Total weight loss
VLCD: Very-low-calorie diet
