# Supplementary material for: Effectiveness of a Low-Calorie Diet for Liver Volume Reduction Prior to Bariatric Surgery: a Systematic Review
Source: Obes Surg. 2020 Nov 2;31(1):350–6. doi: 10.1007/s11695-020-05070-6 (PMC7808983; doi:10.1007/s11695-020-05070-6)
Supplement: Supplementary file 1 — (DOCX 68 kb) [file 11695_2020_5070_MOESM1_ESM.docx]

**Supplementary Table 1.** Search strategy MEDLINE, EMBASE and Cochrane

Search strategy MEDLINE (PubMed)

| #1 | "Bariatric Surgery"[MeSH] OR bariatric surger*[tiab] OR bariatric surgical[tiab] OR metabolic surger*[tiab] OR stomach stapling[tiab] |
| --- | --- |
| #2 | gastric bypass*[tiab] OR gastroileal bypass*[tiab] OR gastrojejunostom*[tiab] |
| #3 | obesty surger*[tiab] |
| #4 | #1 OR #2 OR #3 |
| #5 | "Caloric Restriction"[MeSH] OR calori*[tiab] AND restrict*[tiab] OR low calori*[tiab] OR crash diet*[tiab] OR low energy[tiab] |
| #6 | #4 AND #5 |

Search strategy EMBASE (Ovid)

| 1. | exp bariatric surgery/ |  |
| --- | --- | --- |
| 2. | (bariatric surger* or bariatric surgical or metabolic surger* or stomach stapling).ab,kw,ti. | |
| 3. | (gastric bypass* or gastroileal bypass* or gastrojejunostom*).ab,kw,ti. | |
| 4. | "obesity surger*".ab,kw,ti. | |
| 5. | 1 or 2 or 3 or 4 | |
| 6. | exp caloric restriction/ | |
| 7. | (calori* and restrict*).ab,kw,ti. | |
| 8. | (low calorie* or crash diet or low energy).ab,kw,ti. | |
| 9. | 6 or 7 or 8 | |
| 10. | 5 and 9 | |

Search strategy Cochrane Library

| #1 | (bariatric surger*):ti,ab,kw OR ("bariatric surgical"):ti,ab,kw OR (metabolic surger*):ti,ab,kw OR ("stomach stapling"):ti,ab,kw OR (gastric bypass*):ti,ab,kw |
| --- | --- |
| #2 | (gastric bypass*):ti,ab,kw OR (gastroileal bypass*):ti,ab,kw OR (gastrojejunostom*):ti,ab,kw OR (obesity surger*):ti,ab,kw |
| #3 | #1 OR #2 |
| #4 | (caloric restriction):ti,ab,kw OR (calori* NEAR restrict*):ti,ab,kw OR (low NEAR calorie*):ti,ab,kw OR (crash NEAR diet*):ti,ab,kw OR (low NEAR energy) :ti,ab,kw |
| #5 | #3 AND #4 |

**Supplementary Figure 1**. Risk of bias of RCTs assesed by the Cochrane Collaboration tool

| Author | Selection bias | | Performance bias | Detection bias | Attrition bias | Reporting bias | Other bias | Total |
| --- | --- | --- | --- | --- | --- | --- | --- | --- |
|  | Random sequence generation | Allocation concealment | Blinding of participants and personnel | Blinding of outcome assessment | Incomplete outcome data | Selective reporting |  |  |
| Chakravartty et al. [22] |  |  |  | 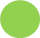 |  |  |  |  |
| Contreras  et al. [12] |  |  | **?** |  |  |  |  |  |
| Bakker et al. [23] |  |  |  |  |  |  |  |  |

*= low risk on bias = moderate risk on bias* **?**  *= unclear*

**Supplementary Table 2**. Study quality of the observational studies assessed by the Modified Downs & Black tool

| **Reporting** | Edholm 2015 [26] | Ekici [24] | González-Pérez [4] | Edholm 2011 [11] | Schiavo [25] |
| --- | --- | --- | --- | --- | --- |
| Is the hypothesis/aim/objective of the study clearly described? | 1 | 1 | 1 | 1 | 1 |
| Are the main outcomes to be measured clearly described in the Introduction or Methods section? | 1 | 1 | 1 | 1 | 1 |
| Are the characteristics of the patients included in the study clearly described? | 1 | 1 | 1 | 1 | 1 |
| Are the interventions of interest clearly described? | 2 | 1 | 2 | 2 | 2 |
| Are the main findings of the study clearly described? | 1 | 1 | 0 | 1 | 1 |
| Does the study provide estimates of the random variability in the data for the main outcomes? | 1 | 1 | 0 | 1 | 1 |
| Have all important adverse events that may be a consequence of the intervention been reported? | 1 | 1 | 1 | 1 | 1 |
| Have the characteristics of patients lost to follow-up been described? | 1 | 1 | 1 | 1 | 0 |
| Have actual probability values been reported (e.g. 0.035 rather than <0.05) for the main outcomes except where the probability value is less than 0.001? | 0 | 1 | 1 | 1 | 1 |
| **External validity** |  |  |  |  |  |
| Were the subjects asked to participate in the study representative of the entire population from which they were recruited? | 0 | U/D | U/D | 0 | 0 |
| Were those subjects who were prepared to participate representative of the entire population from which they were recruited? | 0 | U/D | U/D | 0 | 0 |
| Were the staff, places, and facilities where the patients were treated, representative of the treatment the majority of patients receive? | 1 | 1 | 1 | 1 | 1 |
| **Internal validity - bias** |  |  |  |  |  |
| Was an attempt made to blind study subjects to the intervention they have received? | 0 | 0 | 0 | 0 | 0 |
| Was an attempt made to blind those measuring the main outcomes of the intervention? | 1 | 0 | 0 | 0 | 0 |
| If any of the results of the study were based on “data dredging”, was this made clear? | 0 | 0 | 0 | 0 | 0 |
| In trials and cohort studies, do the analyses adjust for different lengths of follow-up of patients, or in case-control studies, is the time period between the intervention and outcome the same for cases and controls? | 1 | 1 | 1 | 1 | 1 |
| Were the statistical tests used to assess the main outcomes appropriate? | 1 | 1 | 1 | 1 | 1 |
| Was compliance with the intervention/s reliable? | 1 | 1 | 0 | 1 | 1 |
| Were the main outcome measures used accurate (valid and reliable)? | 2 | 1 | 2 | 2 | 1 |
| **Internal validity - confounding (selection bias)** |  |  |  |  |  |
| Were losses of patients to follow-up taken into account? | 1 | 1 | 1 | 1 | U/D |
| Variety in patients? Confounders? Age, weight etc | 1 | 0 | 1 | 1 | 1 |
| **Power** |  |  |  |  |  |
| Did the study have sufficient power to detect a clinically important effect where the probability value for a difference being due to chance is less than 5%? | 0 | 0 | 0 | 0 | 0 |
| Overall | **Fair**  18 | **Fair**  15 | **Fair**  17 | **Fair**  18 | **Fair**  15 |

Abbreviations: U/D = unable to determine.

**Supplementary Table 3.** Results of compliance & tolerance, surgical-, biochemical-, clinical outcomes and complications.

| Author | Compliance & Tolerance | Surgical outcomes | Biochemical and clinical outcomes | Complications | Additional data |
| --- | --- | --- | --- | --- | --- |
| Bakker et al. [23] | Worse compliance due to side effects, like taste and persistent hunger/appetite and constipation compared to control* | N/A | N/A | No difference in incidence of complications | Decreased visceral fat  Decreased skeletal muscle area |
| Chakravartty et al. [22] | N/A | No difference in operating complexity assessed by surgeon.  Similar operating duration, blood loss, operative anatomic scores and hospital stay | N/A | No difference in incidence of complications | No difference in abdominal fat loss  Equal weight loss 3 months post-surgery |
| Contreras et al. [12]  800 & 1200 kcal | High diet adherence in both diets  Worse tolerance in 800 kcal compared to 1200 kcal diet. More dizziness and asthenia in the beginning of the diet. | Same duration of hospital stay comparing both diets | Decrease in BP, improved lipid and glycemic profiles | No difference in incidence of complications between high or low adherence to the LCD | Equal weight loss 6 months post-surgery in both diets  Weight loss by LCD predicted weight loss 6 months post-surgery (1kg loss LCD = 0.79 kg post-surgery) |
| Edholm et al. 2011 [11] | N/A | Decreased operating complexity than control assessed by surgeon. Same omentum size.  Improvement on exposure and amount of psychologic stress induced  No difference in operating time or hospital stay duration. More bleeding in LCD | N/A | No leaks or major postoperative complications occurred | N/A |
| Edholm et al. 2015 [26] | 50% had ketonuria during LCD  Well tolerated, but urge of something to chew increased from day 3 to 28 | N/A | Improved systolic BP, insulin and lipid levels  Unaffected diastolic BP and glucose levels | No complications occurred | Most pronounced weight loss between day 0 and 3 (0.7 kg/day), 51% of this weight loss was accounted by water loss |
| Ekici et al. [24] | 50% of patients did not accept the LCD -> control group  High diet adherence in LCD group (89%)  Four patients ended the diet, they had a small or moderate amount of ketones in urine | Shorter operating duration and hospital stay compared to controls | N/A | No difference in early complication rates between groups | Body weight postoperative was lower at 1, 3, 6, and 12 months post operation |
| González-Pérez et al. [4] | High acceptability (80%)  Discrete frequency of nausea (15%) and diarrhea (15%) and some days of hunger (30%) | N/A | Unaffected HR and BP | N/A | Decreased perimeter of neck, and waist and hip diameter |
| Schiavo et al. [25] | High acceptability (80% agree or strongly agree); Frequently reported hunger (5%) and headache (8%); Occasionally reported intestinal problems (10%)  Total ketone score was highly correlated with the total percentage weight reduction | N/A | Improved glycemic and lipid profile. Unchanged kidney parameters | N/A | Decreased visceral fat |
